# Crystal structure, Hirshfeld surface analysis and DFT studies of 2-[5-(4-methyl­benz­yl)-6-oxo-3-phenyl-1,6-di­hydro­pyridazin-1-yl]acetic acid

**DOI:** 10.1107/S2056989019015317

**Published:** 2019-11-26

**Authors:** Said Daoui, Cemile Baydere, Fouad El Kalai, Lhassane Mahi, Necmi Dege, Khalid Karrouchi, Noureddine Benchat

**Affiliations:** a Laboratory of Applied Chemistry and Environment (LCAE), Faculty of Sciences, Mohamed I University, 60000 Oujda, Morocco; b Department of Physics, Faculty of Arts and Sciences, Ondokuz Mayıs University, 55139-Samsun, Turkey; c Moroccan Foundation for Advanced Science, Innovation and Research (Mascir), Department of Nanotechnology, Rabat Design Center, Rue Mohamed Al Jazouli-Madinat Al Irfane, Rabat 10 100, Morocco; d Laboratory of Plant Chemistry, Organic and Bioorganic Synthesis, URAC23, Faculty of Science, B.P. 1014, GEOPAC Research Center, Mohammed V University, Rabat, Morocco

**Keywords:** crystal structure, hydrogen bonding, DFT, Hirshfeld surface analysis, HOMO–LUMO calculations

## Abstract

The title pyridazinone derivative, C_20_H_18_N_2_O_3_, is not planar. The phenyl ring and the pyridazine ring are inclined to each other by 10.55 (12)°, whereas the 4-methyl­benzyl ring is nearly orthogonal to the pyridazine ring, with a dihedral angle of 72.97 (10)°. In the crystal, mol­ecules are linked by pairs of O—H⋯O hydrogen bonds, forming inversion dimers with an *R*_2_^2^(14) ring motif. The dimers are linked by C—H⋯O hydrogen bonds, generating ribbons propagating along the *c*-axis direction. The inter­molecular inter­actions were additionally investigated using Hirshfeld surface analysis and two-dimensional fingerprint plots. They revealed that the most significant contributions to the crystal packing are from H⋯H (48.4%), H⋯O/O⋯H (21.8%) and H⋯C/C⋯H (20.4%) contacts. Mol­ecular orbital calculations providing electron-density plots of HOMO and LUMO mol­ecular orbitals and mol­ecular electrostatic potentials (MEP) were also computed, both with the DFT/B3LYP/6–311 G++(d,p) basis set.

## Abstract 2

In the title compound, the phenyl and pyridazine rings are inclined to each other by 10.55 (12)°, whereas the 4-methyl­benzyl ring is nearly orthogonal to the pyridazine ring with a dihedral angle of 72.97 (10)°.

## Chemical context   

Pyridazinone derivatives are important biologically active heterocyclic compounds (Dubey *et al.*, 2015 ▸; Akhtar *et al.*, 2016 ▸), which have been the subject of many studies because of their widespread biological activities, such as inflammatory (Barberot *et al.*, 2018 ▸), anti­bacterial (El-Hashash *et al.*, 2014 ▸), anti­depressant (Boukharsa *et al.*, 2016 ▸), anti­hypertensive (Demirayak *et al.*, 2004 ▸), anti-HIV (Li *et al.*, 2013 ▸), anti­convulsant (Partap *et al.*, 2018 ▸), and their use as herbicidal agents (Asif, 2013 ▸). In addition, it has been shown that pyridazinones are good corrosion inhibitors (Chetouani *et al.*, 2003 ▸) and that they can be used as organic extractants of certain metal ions in the aqueous phase (El Kalai *et al.*, 2019*b* ▸).
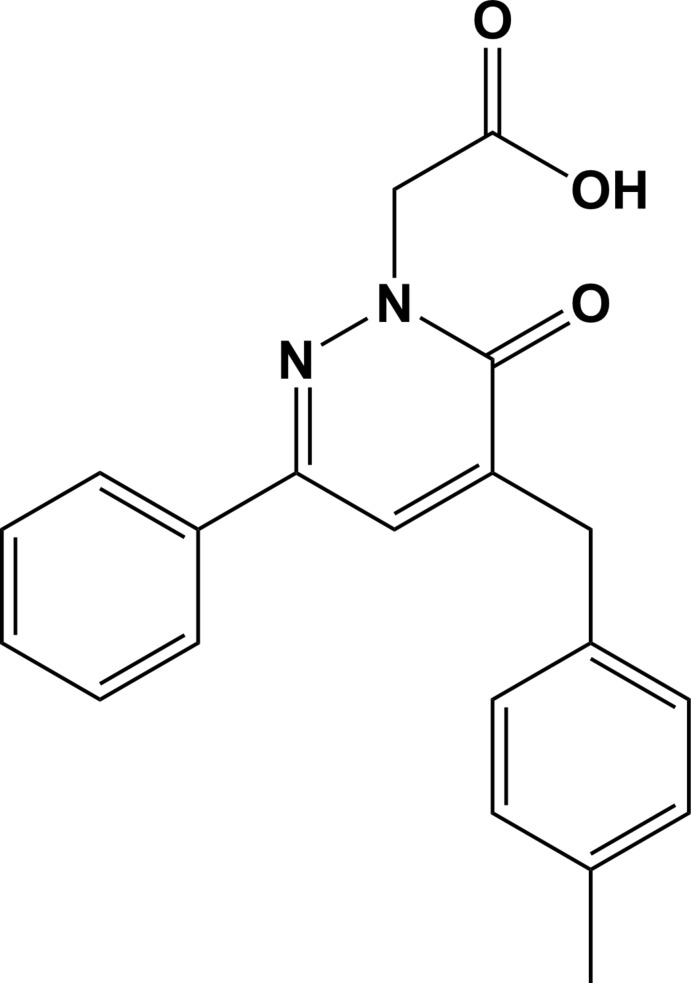


In a continuation of our investigations of the mol­ecular structures and Hirshfeld surfaces of new pyridazinone deriv­atives (Daoui *et al.*, 2019*a* ▸,*b* ▸), we report herein on the synthesis and crystal and mol­ecular structures of the title compound, 2-[5-(4-methyl­benz­yl)-6-oxo-3-phenyl-1,6-di­hydro­pyridazin-1-yl]acetic acid, as well as the analysis of the Hirshfeld surfaces.

## Structural commentary   

**Figure 1:**
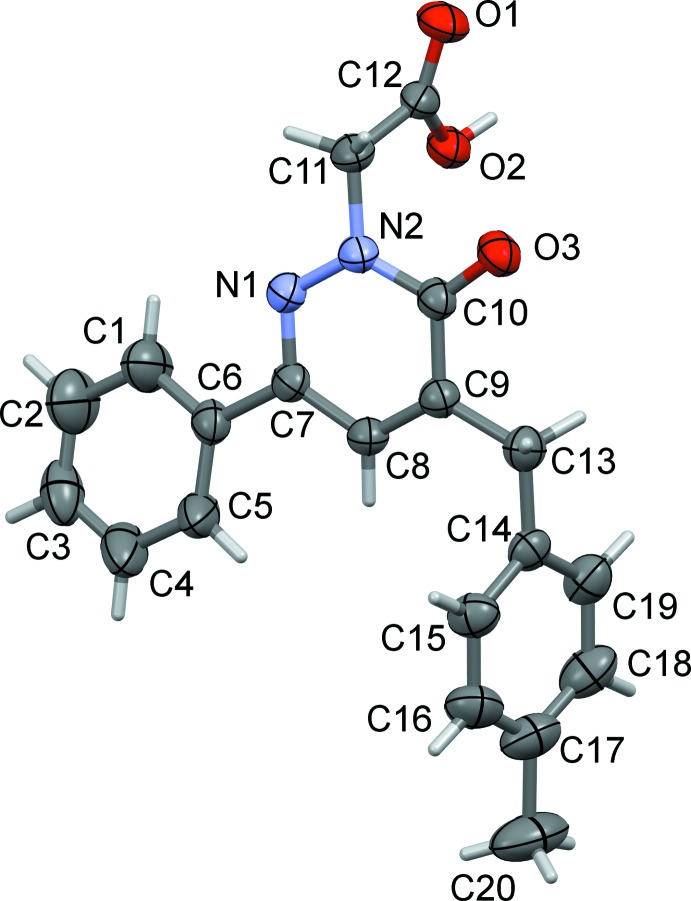
The mol­ecular structure of the title compound, with atom labelling. Displacement ellipsoids are drawn at the 30% probability level.

The mol­ecule structure of the title compound is shown in Fig. 1 ▸. The phenyl (C1–C6) and pyridazine (C7–C10/N1/N2) rings are twisted relative to each other, making a dihedral angle of 10.55 (12)°. The 4-methyl­benzl ring (C14–C19) is inclined to the pyridazine ring by 72.97 (10)°. Atoms C9 and C10 of the pyridazine ring show the largest deviations from planarity (r.m.s. deviation = 0.0075 Å) in positive and negative directions [C10 = 0.0127 (11) Å and C9 = −0.0090 (11) Å]. The O3=C10 bond length of the pyridaz­in­one carbonyl function is 1.2433 (19) Å and the N1—N2 bond length in the pyridazine ring is 1.3516 (19) Å, both in accordance with values reported for related pyridazinones (El Kalai *et al.*, 2019*a* ▸; Xu *et al.*, 2005 ▸).

## Supra­molecular features   

**Figure 2:**
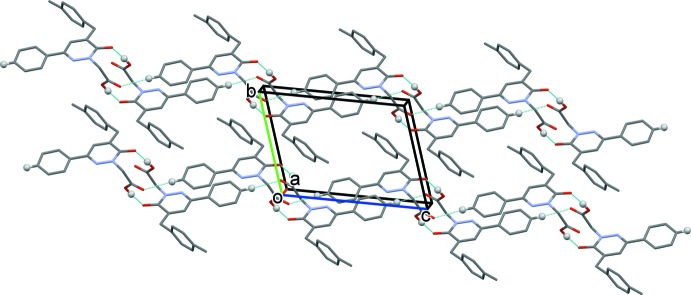
A view along the *a* axis of the crystal packing of the title compound. The O—H⋯O and C—H⋯O hydrogen bonds (see Table 1 ▸) are shown as dashed lines. For clarity, only H atoms H2 and H3 (grey balls) have been included.

In the crystal, mol­ecules are linked by pairs of O—H⋯O hydrogen bonds, forming inversion dimers with an 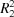
(14) ring motif (Table 1 ▸ and Fig. 2 ▸). The dimers are linked by C—H⋯O hydrogen bonds, forming ribbons that extend along the *c*-axis direction (Table 1 ▸ and Fig. 2 ▸). There are no other significant inter­molecular inter­actions present.

## Database survey   

A search of the Cambridge Structural Database (CSD, version 5.40, update August 2019; Groom *et al.*, 2016 ▸) using 2-[6-oxopyridazin-1(6*H*)-yl]acetic acid as the main skeleton revealed the presence of six structures similar to the title compound, but with different substituents on the pyridazine ring. Two of these structures are similar to the title compound, *viz.* ethyl {5-[(3-chloro­phen­yl)meth­yl]-6-oxo-3-phenyl­pyrid­azin-1(6*H*)-yl}acetate (FODQUN; El Kalai *et al.*, 2019*a* ▸) and ethyl 3-methyl-6-oxo-5-[3-(tri­fluoro­meth­yl)phen­yl]-1,6-di­hydro-1-pyridazine­acetate (QANVOR; Xu *et al.*, 2005 ▸).

In FODQUN, the phenyl ring and the pyridazine ring are inclined to each other by 17.41 (13)°, whereas the 3-chloro­phenyl ring is nearly orthogonal to the pyridazine ring with a dihedral angle of 88.19 (13)°. In the crystal, C—H⋯O hydrogen bonds generate inversion dimers with an 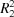
(10) ring motif. The dimers are linked by further C—H⋯O hydrogen bonds, enclosing *R*_2_^2^(20) ring motifs, forming ribbons, similar to the situation in the crystal of the title compound. Weak inter­molecular C—H⋯π inter­actions and π–π inter­actions are also observed in the crystal structure.

In QANVOR, the phenyl and pyridazinone rings are approximately coplanar with a dihedral angle of 4.84 (14)°. In the crystal, inversion-related mol­ecules form dimers through non-classical C—H⋯O hydrogen bonds. The dimers are linked by a number of C–H⋯F hydrogen bonds, forming a three-dimensional structure.

## Hirshfeld surface analysis   

**Figure 3:**
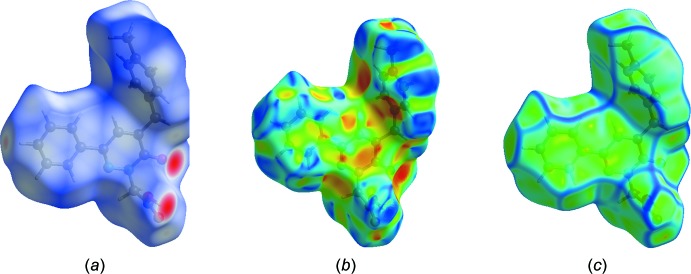
(*a*) The Hirshfeld surface of the title compound mapped over *d*_norm_, and plotted in the range −0.7290 to 1.4764 a.u.. (*b*) the Hirshfeld surface mapped over shape-index, (*c*) the Hirshfeld surface mapped over curvedness.

Hirshfeld surface analysis was used to qu­antify the inter­molecular contacts of the title compounds, using the software *CrystalExplorer17.5* (Turner *et al.*, 2017 ▸). The Hirshfeld surfaces were calculated using a standard (high) surface resolution with the three-dimensional *d*_norm_ surfaces plotted over a fixed colour scale of −0.7290 (red) to 1.4764 (blue) a.u.. The Hirshfeld surfaces of the title compound were mapped over *d*_norm_, shape index and curvedness, and are shown in Fig. 3 ▸ *a*–*c*.

**Figure 4:**
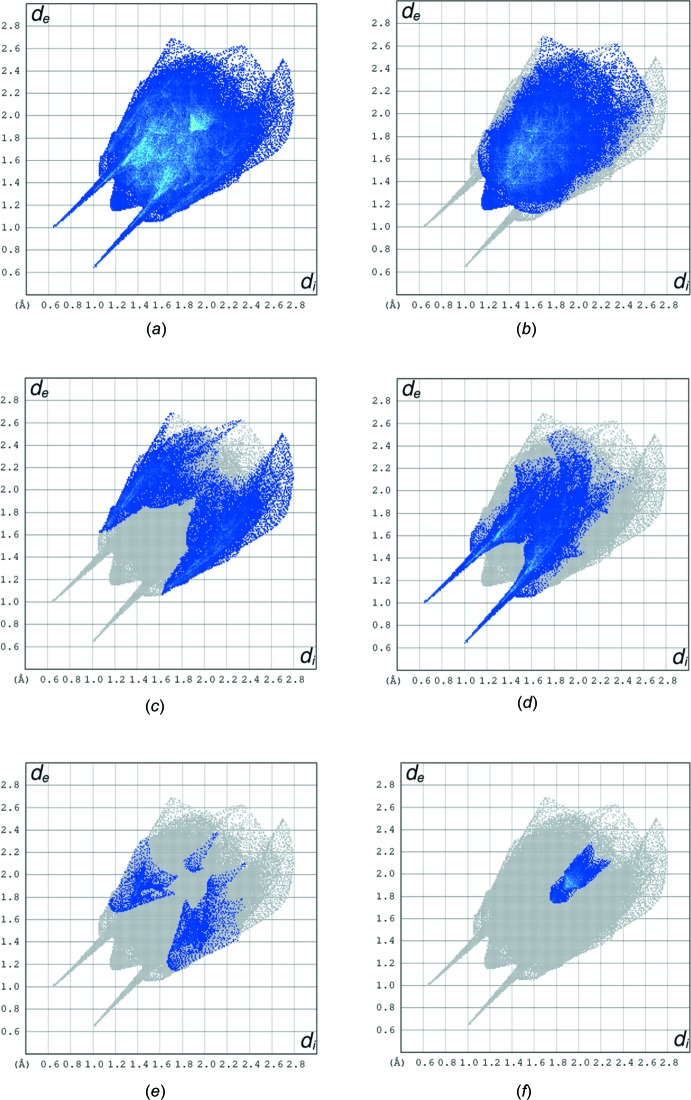
(*a*) The full two-dimensional fingerprint plot for the title compound, and delineated into (*b*) H⋯H (48.4%), (*c*) H⋯C/C⋯H (20.4%), (*d*) H⋯O/O⋯H (21.8%), (*e*) H⋯N/N⋯H (4.1%) and (*f*) C⋯C (4.0%) contacts.

The overall two-dimensional fingerprint plot and those delineated into H⋯H, H⋯C/ C⋯H, H⋯O/O⋯H, H⋯N/N⋯H and C⋯C contacts are illustrated in Fig. 4 ▸ *a*–*f*, respectively. The H⋯H inter­action makes the largest contribution (48.4%) to the overall crystal packing. The pair of wings in the fingerprint plot delineated into H⋯C/C⋯H contacts, which contribute 20.4% to the Hirshfeld surface, have a nearly symmetrical distribution of points with the tips at *d*_e_ + *d*_i_ ∼2.70 Å. H⋯O/O⋯H contacts make a 21.8% contribution to the Hirshfeld surface. The contacts are represented by a pair of sharp spikes in the region *d*_e_ + *d*_i_ ∼1.64 Å in the fingerprint plot, Fig. 4 ▸ *d*. The H⋯O/O⋯H contacts arise from inter­molecular O—H⋯O and C—H⋯O hydrogen bonding (Table 2 ▸). The contributions of the other contacts to the Hirshfeld surface are negligible, *i.e*. H⋯N/N⋯H of 4.1% and C⋯C of 4.0%.

## Frontier mol­ecular orbital analysis   

**Figure 5:**
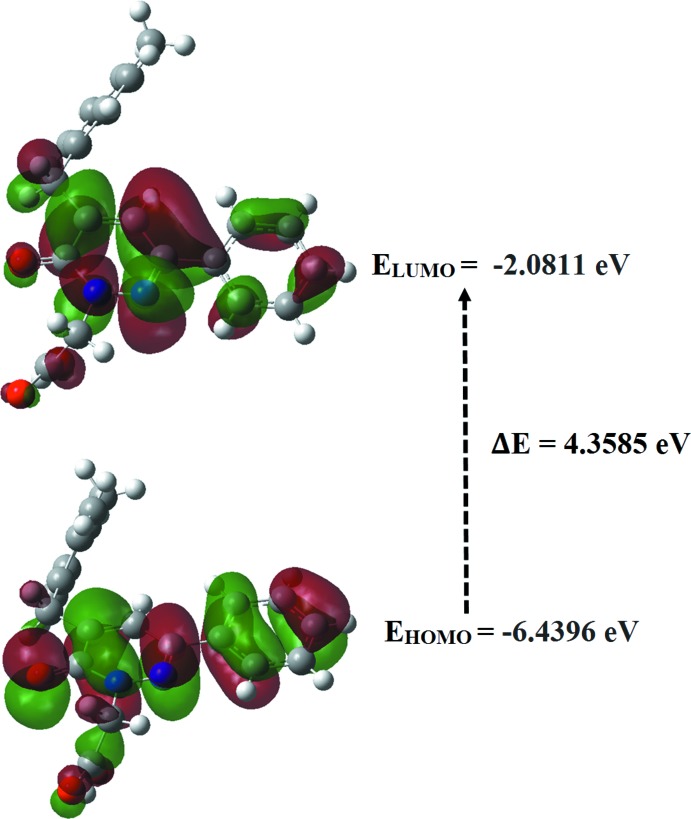
Mol­ecular orbital energy levels of the title compound.

The energy levels for the title compound were computed theoretically *via* density functional theory (DFT) using the standard B3LYP functional and 6–311 G++ (d,p) basis-set calculations (Becke, 1993 ▸) as implemented in *GAUSSIAN 09* (Frisch *et al.*, 2009 ▸). The HOMO (highest occupied mol­ecular orbital) acts as an electron donor and the LUMO (lowest occupied mol­ecular orbital) as an electron acceptor. When the energy gap is small, the mol­ecule is highly polarizable and has high chemical reactivity. The energy levels, energy gaps, hardness (η), softness (σ) and electronegativity (χ) are given in Table 2 ▸. The electron transition from the HOMO to the LUMO energy level is shown in Fig. 5 ▸. The chemical hardness and softness of a mol­ecule is a sign of its chemical stability. From the HOMO–LUMO energy gap, we can see whether or not the mol­ecule is hard or soft. If the energy gap is large, the mol­ecule is hard and if small the mol­ecule is soft. Soft mol­ecules are more polarizable than hard ones because they need less energy for excitation. Therefore, from Table 2 ▸ we conclude that the title compound can be classified as a hard material with a HOMO–LUMO energy gap of 4.3585 eV.

## Mol­ecular electrostatic potentials   

**Figure 6:**
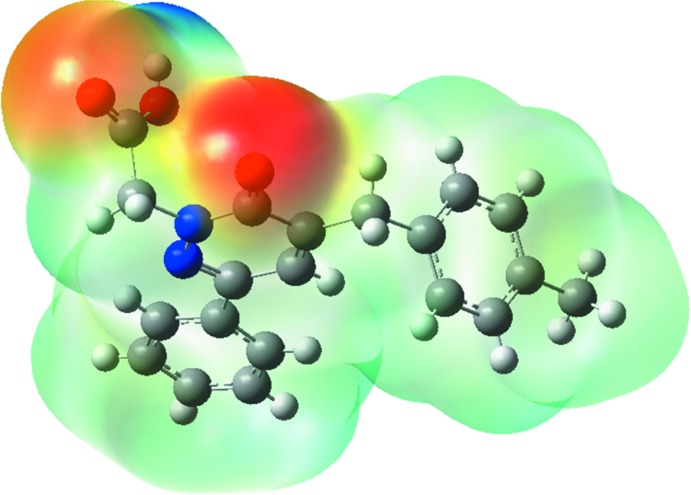
Theoretical mol­ecular electrostatic potential surface for the title compound, calculated using the DFT/B3LYP/6–311 G++ (d,p) basis set.

Mol­ecular electrostatic potential (MEP) displays mol­ecular size and shape as well as positive, negative and neutral electrostatic potential regions in terms of colour grading and is useful in investigating relationships between mol­ecular structure and physicochemical properties (Murray & Sen, 1996 ▸; Scrocco & Tomasi, 1978 ▸). The MEP map (Fig. 6 ▸) was calculated at the B3LYP/6-311 G++ (d,p) level of theory. The red and blue-coloured regions indicate nucleophiles (electron rich) and electrophile regions (electron poor), respectively. The white regions indicate neutral atoms. In the title mol­ecule, the red regions are concentrated at the carbonyl group. It possesses the most negative potential and is thus the strongest repulsion site (electrophilic attack). The blue regions indicate the strongest attraction regions, which are occupied mostly by hydrogen atoms.

## Synthesis and crystallization   

A suspension of ethyl 2-[5-(4-methyl­benz­yl)-6-oxo-3-phenyl­pyridazin-1(6*H*)-yl]acetate (3.6 mmol), and 6 *N* NaOH (14.4 mmol) in ethanol (50 ml) was stirred at 353 K for 4 h. The mixture was then concentrated *in vacuo*, diluted with cold water, and acidified with 6 *N* HCl. The final product was filtered off with suction and recrystallized from ethanol. Yellow prismatic crystals were obtained by slow evaporation of the solvent at room temperature.

## Refinement   

Crystal data, data collection and structure refinement details are summarized in Table 3 ▸. The hydrogen atoms were fixed geometrically (O—H = 0.82 Å, C—H = 0.93–0.96 Å) and allowed to ride on their parent atoms with *U*_iso_(H) = 1.5*U*_eq_(O, C-meth­yl) and 1.2*U*_eq_(C) for other H atoms. For atoms C17–C20, SIMU, DELU and ISOR commands were used (*SHELXL*; Sheldrick, 2015*b* ▸).

## Supplementary Material

Crystal structure: contains datablock(s) I, Global. DOI: 10.1107/S2056989019015317/su5527sup1.cif

Structure factors: contains datablock(s) I. DOI: 10.1107/S2056989019015317/su5527Isup2.hkl

Supporting information file. DOI: 10.1107/S2056989019015317/su5527Isup3.cml

CCDC reference: 1965448

Additional supporting information:  crystallographic information; 3D view; checkCIF report

## Figures and Tables

**Table 1:** Hydrogen-bond geometry (Å, °)

*D*—H⋯*A*	*D*—H	H⋯*A*	*D*⋯*A*	*D*—H⋯*A*
O2—H2⋯O3^i^	0.82	1.83	2.6358 (16)	167
C3—H3⋯O1^ii^	0.93	2.51	3.430 (3)	172

Symmetry codes: (i) 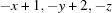; (ii) 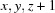.

**Table 2:** Calculated frontier mol­ecular orbital energies (eV)

FMO	Energy
*E*(HOMO)	–6.4396
*E*(LUMO)	–2.0811
Δ*E*(HOMO–LUMO)	4.3585
Hardness, η	2.1792
Softness, σ	0.4589
Electronegativity, χ	4.2603

**Table 3:** Experimental details

Crystal data	
Chemical formula	C_20_H_18_N_2_O_3_
*M*_r_	334.36
Crystal system, space group	Triclinic, *P* 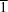
Temperature (K)	296
*a*, *b*, *c* (Å)	8.4213 (7), 9.0739 (9), 12.2238 (12)
α, β, γ (°)	106.501 (8), 92.390 (8), 100.750 (8)
*V* (Å^3^)	875.43 (15)
*Z*	2
Radiation type	Mo *K*α
μ (mm^−1^)	0.09
Crystal size (mm)	0.75 × 0.62 × 0.34

Data collection	
Diffractometer	Stoe *IPDS* 2
Absorption correction	Integration (*X-RED32*; Stoe & Cie, 2002 ▸)
*T*_min_, *T*_max_	0.945, 0.959
No. of measured, independent and observed [*I* > 2σ(*I*)] reflections	7687, 3387, 2159
*R*_int_	0.029
(sin θ/λ)_max_ (Å^−1^)	0.617

Refinement	
*R*[*F* ^2^ > 2σ(*F* ^2^)], *wR*(*F* ^2^), *S*	0.046, 0.130, 1.01
No. of reflections	3387
No. of parameters	228
No. of restraints	33
H-atom treatment	H-atom parameters constrained
Δρ_max_, Δρ_min_ (e Å^−3^)	0.12, −0.14

Computer programs: *X-AREA* and *X-RED32* (Stoe & Cie, 2002 ▸), *SHELXT2017* (Sheldrick, 2015*a* ▸), *SHELXL2018* (Sheldrick, 2015*b* ▸), *Mercury* (Macrae *et al.*, 2008 ▸), *WinGX* (Farrugia, 2012 ▸), *PLATON* (Spek, 2009 ▸) and *publCIF* (Westrip, 2010 ▸).

## supplementary crystallographic information

### Crystal data

**Table:**

C_20_H_18_N_2_O_3_	*Z* = 2
*M**_r_* = 334.36	*F*(000) = 352
Triclinic, *P*1	*D*_x_ = 1.268 Mg m^−^^3^
*a* = 8.4213 (7) Å	Mo *K*α radiation, λ = 0.71073 Å
*b* = 9.0739 (9) Å	Cell parameters from 9277 reflections
*c* = 12.2238 (12) Å	θ = 2.4–30.5°
α = 106.501 (8)°	µ = 0.09 mm^−^^1^
β = 92.390 (8)°	*T* = 296 K
γ = 100.750 (8)°	Prism, yellow
*V* = 875.43 (15) Å^3^	0.75 × 0.62 × 0.34 mm

### Data collection

**Table:**

Stoe IPDS 2 diffractometer	3387 independent reflections
Radiation source: sealed X-ray tube, 12 x 0.4 mm long-fine focus	2159 reflections with *I* > 2σ(*I*)
Plane graphite monochromator	*R*_int_ = 0.029
Detector resolution: 6.67 pixels mm^-1^	θ_max_ = 26.0°, θ_min_ = 2.5°
rotation method scans	*h* = −10→10
Absorption correction: integration (X-RED32; Stoe & Cie, 2002)	*k* = −10→11
*T*_min_ = 0.945, *T*_max_ = 0.959	*l* = −15→15
7687 measured reflections	

### Refinement

**Table:**

Refinement on *F*^2^	Primary atom site location: dual
Least-squares matrix: full	Secondary atom site location: difference Fourier map
*R*[*F*^2^ > 2σ(*F*^2^)] = 0.046	Hydrogen site location: inferred from neighbouring sites
*wR*(*F*^2^) = 0.130	H-atom parameters constrained
*S* = 1.01	*w* = 1/[σ^2^(*F*_o_^2^) + (0.0706*P*)^2^] where *P* = (*F*_o_^2^ + 2*F*_c_^2^)/3
3387 reflections	(Δ/σ)_max_ < 0.001
228 parameters	Δρ_max_ = 0.12 e Å^−^^3^
33 restraints	Δρ_min_ = −0.14 e Å^−^^3^

### Special details

**Table:**

Geometry. All esds (except the esd in the dihedral angle between two l.s. planes) are estimated using the full covariance matrix. The cell esds are taken into account individually in the estimation of esds in distances, angles and torsion angles; correlations between esds in cell parameters are only used when they are defined by crystal symmetry. An approximate (isotropic) treatment of cell esds is used for estimating esds involving l.s. planes.

### Fractional atomic coordinates and isotropic or equivalent isotropic displacement parameters (Å^2^)

**Table:**

	*x*	*y*	*z*	*U*_iso_*/*U*_eq_	
O2	0.63260 (15)	1.10394 (16)	0.10937 (10)	0.0735 (4)	
H2	0.597450	1.162100	0.078353	0.110*	
O3	0.52647 (15)	0.73205 (16)	−0.01452 (10)	0.0764 (4)	
O1	0.80943 (17)	1.09371 (19)	−0.02162 (12)	0.0945 (5)	
N2	0.68471 (16)	0.84641 (18)	0.15268 (12)	0.0669 (4)	
N1	0.72008 (16)	0.88379 (18)	0.26737 (12)	0.0668 (4)	
C8	0.46392 (19)	0.7210 (2)	0.26864 (14)	0.0620 (4)	
H8	0.389598	0.679405	0.311801	0.074*	
C9	0.4288 (2)	0.6836 (2)	0.15452 (14)	0.0620 (4)	
C7	0.61225 (19)	0.8228 (2)	0.32473 (14)	0.0613 (4)	
C14	0.1506 (2)	0.5207 (2)	0.15768 (15)	0.0664 (4)	
C6	0.6544 (2)	0.8672 (2)	0.45037 (15)	0.0680 (5)	
C10	0.5447 (2)	0.7524 (2)	0.09055 (14)	0.0634 (4)	
C12	0.7494 (2)	1.0492 (2)	0.05303 (14)	0.0670 (5)	
C13	0.2753 (2)	0.5779 (2)	0.08692 (16)	0.0734 (5)	
H13A	0.226930	0.634241	0.042417	0.088*	
H13B	0.303827	0.487485	0.033334	0.088*	
C11	0.8048 (2)	0.9243 (3)	0.09364 (17)	0.0773 (5)	
H11A	0.830690	0.846172	0.028180	0.093*	
H11B	0.903514	0.971292	0.145133	0.093*	
C15	0.1700 (3)	0.4072 (2)	0.20852 (19)	0.0832 (6)	
H15	0.258956	0.359741	0.194728	0.100*	
C19	0.0148 (3)	0.5824 (2)	0.1768 (2)	0.0885 (6)	
H19	−0.003886	0.657256	0.142058	0.106*	
C17	−0.0729 (3)	0.4267 (3)	0.3010 (2)	0.1019 (7)	
C18	−0.0948 (3)	0.5352 (3)	0.2469 (2)	0.1060 (7)	
H18	−0.186687	0.578672	0.257801	0.127*	
C16	0.0607 (3)	0.3626 (3)	0.2792 (2)	0.0992 (7)	
H16	0.078355	0.286573	0.313098	0.119*	
C3	0.7361 (4)	0.9592 (4)	0.6852 (2)	0.1101 (9)	
H3	0.763972	0.989204	0.763999	0.132*	
C1	0.7831 (3)	0.9861 (3)	0.5033 (2)	0.1070 (8)	
H1	0.844994	1.038827	0.459306	0.128*	
C5	0.5672 (3)	0.7970 (4)	0.51898 (19)	0.1116 (9)	
H5	0.478402	0.715558	0.486763	0.134*	
C4	0.6081 (3)	0.8448 (5)	0.6369 (2)	0.1338 (11)	
H4	0.545236	0.796070	0.682551	0.161*	
C2	0.8236 (4)	1.0299 (4)	0.6197 (2)	0.1281 (10)	
H2A	0.913185	1.110017	0.653001	0.154*	
C20	−0.1911 (4)	0.3793 (4)	0.3811 (3)	0.1591 (13)	
H20A	−0.271221	0.443328	0.391357	0.239*	
H20B	−0.133254	0.393504	0.453929	0.239*	
H20C	−0.243577	0.270862	0.348701	0.239*	

### Atomic displacement parameters (Å^2^)

**Table:**

	*U*^11^	*U*^22^	*U*^33^	*U*^12^	*U*^13^	*U*^23^
O2	0.0725 (8)	0.0961 (10)	0.0601 (7)	0.0214 (7)	0.0150 (6)	0.0325 (6)
O3	0.0785 (8)	0.1010 (10)	0.0580 (7)	0.0288 (7)	0.0140 (6)	0.0293 (7)
O1	0.0939 (10)	0.1310 (12)	0.0796 (9)	0.0267 (9)	0.0331 (8)	0.0589 (9)
N2	0.0532 (8)	0.0909 (10)	0.0664 (9)	0.0154 (7)	0.0119 (7)	0.0379 (8)
N1	0.0535 (8)	0.0864 (10)	0.0657 (9)	0.0138 (7)	0.0053 (7)	0.0312 (8)
C8	0.0572 (9)	0.0719 (11)	0.0606 (10)	0.0117 (8)	0.0105 (8)	0.0260 (8)
C9	0.0593 (9)	0.0686 (10)	0.0612 (10)	0.0155 (8)	0.0085 (8)	0.0226 (8)
C7	0.0534 (9)	0.0744 (11)	0.0620 (10)	0.0180 (8)	0.0085 (8)	0.0263 (8)
C14	0.0630 (10)	0.0584 (10)	0.0684 (10)	0.0029 (8)	0.0007 (8)	0.0111 (8)
C6	0.0595 (10)	0.0875 (12)	0.0599 (10)	0.0225 (9)	0.0044 (8)	0.0219 (9)
C10	0.0600 (10)	0.0788 (11)	0.0586 (10)	0.0231 (8)	0.0098 (8)	0.0257 (8)
C12	0.0584 (10)	0.0908 (13)	0.0515 (9)	0.0060 (9)	0.0068 (8)	0.0267 (9)
C13	0.0737 (11)	0.0748 (11)	0.0642 (10)	0.0050 (9)	−0.0002 (9)	0.0165 (9)
C11	0.0556 (9)	0.1085 (15)	0.0800 (12)	0.0164 (10)	0.0190 (9)	0.0462 (11)
C15	0.0817 (13)	0.0735 (12)	0.0977 (14)	0.0177 (10)	0.0199 (11)	0.0284 (11)
C19	0.0741 (13)	0.0770 (13)	0.1100 (17)	0.0140 (10)	0.0034 (12)	0.0228 (12)
C17	0.0866 (14)	0.0909 (14)	0.1061 (16)	−0.0089 (12)	0.0287 (11)	0.0092 (10)
C18	0.0733 (12)	0.1004 (16)	0.1320 (19)	0.0157 (12)	0.0256 (13)	0.0142 (12)
C16	0.1108 (18)	0.0792 (14)	0.1074 (17)	0.0029 (13)	0.0224 (14)	0.0366 (13)
C3	0.1037 (19)	0.164 (3)	0.0617 (13)	0.0585 (19)	0.0004 (14)	0.0139 (16)
C1	0.1183 (19)	0.1081 (17)	0.0751 (14)	−0.0077 (15)	−0.0081 (13)	0.0191 (12)
C5	0.0720 (13)	0.189 (3)	0.0717 (13)	−0.0062 (14)	−0.0022 (11)	0.0583 (15)
C4	0.0910 (17)	0.243 (4)	0.0762 (15)	0.023 (2)	0.0094 (14)	0.068 (2)
C2	0.143 (2)	0.133 (2)	0.0775 (17)	0.0047 (19)	−0.0182 (17)	0.0019 (16)
C20	0.138 (2)	0.158 (3)	0.145 (3)	−0.033 (2)	0.064 (2)	0.022 (2)

### Geometric parameters (Å, º)

**Table:**

O2—C12	1.317 (2)	C11—H11B	0.9700
O2—H2	0.8200	C15—C16	1.372 (3)
O3—C10	1.2433 (19)	C15—H15	0.9300
O1—C12	1.193 (2)	C19—C18	1.377 (3)
N2—N1	1.3516 (19)	C19—H19	0.9300
N2—C10	1.370 (2)	C17—C16	1.363 (3)
N2—C11	1.459 (2)	C17—C18	1.367 (4)
N1—C7	1.307 (2)	C17—C20	1.515 (4)
C8—C9	1.344 (2)	C18—H18	0.9300
C8—C7	1.422 (2)	C16—H16	0.9300
C8—H8	0.9300	C3—C2	1.327 (4)
C9—C10	1.438 (2)	C3—C4	1.330 (4)
C9—C13	1.507 (2)	C3—H3	0.9300
C7—C6	1.482 (2)	C1—C2	1.374 (3)
C14—C19	1.365 (3)	C1—H1	0.9300
C14—C15	1.375 (3)	C5—C4	1.390 (3)
C14—C13	1.499 (3)	C5—H5	0.9300
C6—C5	1.352 (3)	C4—H4	0.9300
C6—C1	1.366 (3)	C2—H2A	0.9300
C12—C11	1.498 (3)	C20—H20A	0.9600
C13—H13A	0.9700	C20—H20B	0.9600
C13—H13B	0.9700	C20—H20C	0.9600
C11—H11A	0.9700		
			
C12—O2—H2	109.5	H11A—C11—H11B	107.7
N1—N2—C10	126.14 (14)	C16—C15—C14	121.4 (2)
N1—N2—C11	115.10 (15)	C16—C15—H15	119.3
C10—N2—C11	118.61 (14)	C14—C15—H15	119.3
C7—N1—N2	117.30 (14)	C14—C19—C18	120.8 (2)
C9—C8—C7	121.38 (16)	C14—C19—H19	119.6
C9—C8—H8	119.3	C18—C19—H19	119.6
C7—C8—H8	119.3	C16—C17—C18	116.7 (2)
C8—C9—C10	117.95 (16)	C16—C17—C20	121.4 (3)
C8—C9—C13	125.62 (16)	C18—C17—C20	121.9 (3)
C10—C9—C13	116.42 (15)	C17—C18—C19	122.2 (2)
N1—C7—C8	121.32 (15)	C17—C18—H18	118.9
N1—C7—C6	115.86 (15)	C19—C18—H18	118.9
C8—C7—C6	122.82 (16)	C17—C16—C15	121.7 (2)
C19—C14—C15	117.17 (19)	C17—C16—H16	119.1
C19—C14—C13	121.17 (18)	C15—C16—H16	119.1
C15—C14—C13	121.63 (17)	C2—C3—C4	119.2 (2)
C5—C6—C1	116.45 (19)	C2—C3—H3	120.4
C5—C6—C7	122.76 (18)	C4—C3—H3	120.4
C1—C6—C7	120.77 (19)	C6—C1—C2	121.7 (3)
O3—C10—N2	119.17 (16)	C6—C1—H1	119.2
O3—C10—C9	124.97 (17)	C2—C1—H1	119.2
N2—C10—C9	115.86 (14)	C6—C5—C4	121.1 (3)
O1—C12—O2	124.94 (17)	C6—C5—H5	119.5
O1—C12—C11	121.98 (17)	C4—C5—H5	119.5
O2—C12—C11	113.06 (14)	C3—C4—C5	120.8 (3)
C14—C13—C9	114.91 (15)	C3—C4—H4	119.6
C14—C13—H13A	108.5	C5—C4—H4	119.6
C9—C13—H13A	108.5	C3—C2—C1	120.7 (3)
C14—C13—H13B	108.5	C3—C2—H2A	119.7
C9—C13—H13B	108.5	C1—C2—H2A	119.7
H13A—C13—H13B	107.5	C17—C20—H20A	109.5
N2—C11—C12	113.59 (14)	C17—C20—H20B	109.5
N2—C11—H11A	108.8	H20A—C20—H20B	109.5
C12—C11—H11A	108.8	C17—C20—H20C	109.5
N2—C11—H11B	108.8	H20A—C20—H20C	109.5
C12—C11—H11B	108.8	H20B—C20—H20C	109.5
			
C10—N2—N1—C7	1.2 (2)	C10—C9—C13—C14	−174.43 (15)
C11—N2—N1—C7	176.75 (15)	N1—N2—C11—C12	−107.68 (17)
C7—C8—C9—C10	−1.3 (2)	C10—N2—C11—C12	68.2 (2)
C7—C8—C9—C13	−179.66 (16)	O1—C12—C11—N2	−159.10 (18)
N2—N1—C7—C8	0.1 (2)	O2—C12—C11—N2	22.1 (2)
N2—N1—C7—C6	−179.55 (14)	C19—C14—C15—C16	−2.6 (3)
C9—C8—C7—N1	0.0 (3)	C13—C14—C15—C16	175.73 (19)
C9—C8—C7—C6	179.63 (15)	C15—C14—C19—C18	1.8 (3)
N1—C7—C6—C5	−170.3 (2)	C13—C14—C19—C18	−176.5 (2)
C8—C7—C6—C5	10.0 (3)	C16—C17—C18—C19	−2.2 (4)
N1—C7—C6—C1	11.5 (3)	C20—C17—C18—C19	178.0 (2)
C8—C7—C6—C1	−168.13 (19)	C14—C19—C18—C17	0.6 (4)
N1—N2—C10—O3	177.73 (15)	C18—C17—C16—C15	1.4 (4)
C11—N2—C10—O3	2.3 (2)	C20—C17—C16—C15	−178.7 (3)
N1—N2—C10—C9	−2.4 (2)	C14—C15—C16—C17	0.9 (4)
C11—N2—C10—C9	−177.82 (15)	C5—C6—C1—C2	1.2 (4)
C8—C9—C10—O3	−177.84 (16)	C7—C6—C1—C2	179.5 (2)
C13—C9—C10—O3	0.7 (3)	C1—C6—C5—C4	−0.1 (4)
C8—C9—C10—N2	2.3 (2)	C7—C6—C5—C4	−178.3 (2)
C13—C9—C10—N2	−179.12 (15)	C2—C3—C4—C5	1.2 (5)
C19—C14—C13—C9	104.4 (2)	C6—C5—C4—C3	−1.2 (5)
C15—C14—C13—C9	−73.8 (2)	C4—C3—C2—C1	0.0 (5)
C8—C9—C13—C14	4.0 (3)	C6—C1—C2—C3	−1.2 (5)

### Hydrogen-bond geometry (Å, º)

**Table:**

*D*—H···*A*	*D*—H	H···*A*	*D*···*A*	*D*—H···*A*
O2—H2···O3^i^	0.82	1.83	2.6358 (16)	167
C3—H3···O1^ii^	0.93	2.51	3.430 (3)	172

Symmetry codes: (i) −*x*+1, −*y*+2, −*z*; (ii) *x*, *y*, *z*+1.
